# Rapid Quantification of the Binocular Visual Field for Clinical Trials: Performance of a Modified Esterman Supra-Threshold Test Implemented with the Open Perimetry Interface

**DOI:** 10.2147/OPTH.S352004

**Published:** 2022-05-19

**Authors:** Colm D Andrews, Aislin A Sheldon, Holly Bridge, Susan M Downes, Robert E MacLaren, Jasleen K Jolly

**Affiliations:** 1 Nuffield Department of Population Health, University of Oxford, Oxford, UK; 2 Oxford Eye Hospital, Oxford University Hospitals NHS Foundation Trust, Oxford, UK; 3 Oxford Centre for Functional MRI of the Brain (FMRIB), Wellcome Centre for Integrative Neuroimaging, University of Oxford, Oxford, UK; 4 Nuffield Laboratory of Ophthalmology, Nuffield Department of Clinical Neurosciences, University of Oxford, Oxford, UK; 5 Vision and Eye Research Institute, Anglia Ruskin University, Cambridge, UK

**Keywords:** visual field, functional testing, screening, Stargardt disease, choroideremia

## Abstract

**Purpose:**

We aimed to assess the performance of the modified-Esterman test (mET) as a rapid suprathreshold binocular quantification tool for the assessment of peripheral visual fields. The mET consists of an even spread of test points across the visual field.

**Materials and Methods:**

The mET was implemented on the Octopus 0900 perimeter using the Open Perimetry Interface (OPI) and consisted of 160 points. Patients with choroideremia, a rod-cone dystrophy, Stargardt disease, a cone-rod dystrophy, and healthy volunteers underwent both the mET and the standard Esterman tests twice. Disease severity (mild/moderate/severe) was graded on both tests independently. Voronoi tessellation was utilised to compare the tests.

**Results:**

The Voronoi visualisation was able to demonstrate that the mET was able to provide more information about the disease state at all stages of diseases. This was confirmed by the agreement statistic, which showed that the mET detected 27% more points of visual field loss compared to the Esterman test, being most useful in patients with rod-cone dystrophies.

**Conclusion:**

The mET provides a speedy quantitative measure of the peripheral visual field loss, which can be used in clinical trials to monitor longitudinal assessment of peripheral visual function. The mET provides a more even coverage across the visual field compared to the Esterman test points, making it more suitable for this purpose. This is a key part of safety monitoring in retinal clinical trials. The mET can easily be implemented on commercially available perimeters that allow Open Perimetry.

## Video abstract

**Figure:**
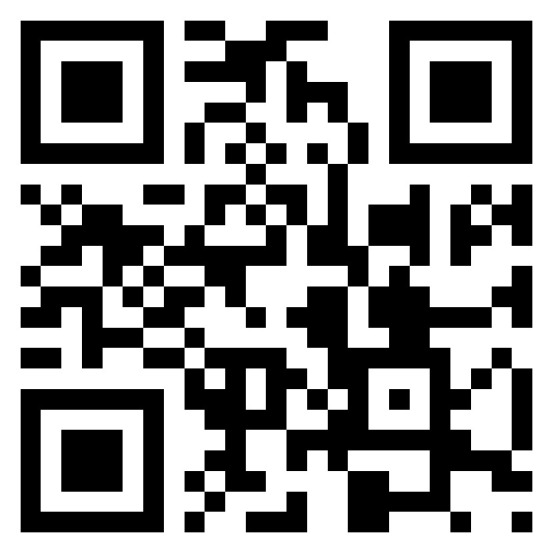

Point your SmartPhone at the code above. If you have a QR code reader the video abstract will appear. Or use:

https://youtu.be/MIfb8AWnkLg

## Introduction

Visual field testing is a key part of the assessment of visual function, because visual field loss has a large impact on patients’ quality of life.^1^ In particular, the peripheral visual field has a key role in several aspects of vision, such as mobility, and cannot be predicted from measuring the central visual field or with standard visual acuity tests.^2^ The visual field as a whole reflects function of a much larger area of the retina than visual acuity and contrast sensitivity, and therefore provides an important complementary measure.

For novel therapy accreditation from the U.S. Food and Drug Administration and other regulatory authorities, certain assessment criteria must be met, and there is a strong emphasis on safety assessment of any therapy.^3^ Functional outcomes are preferred to structural measures and the loss of peripheral visual field has been highlighted as a key component for both safety and effectivity monitoring.^4^

There are several clinical conditions that are monitored through the use of visual field testing in clinical practice, and these include glaucoma and inherited retinal diseases. However, most visual field tests focus on the central 10 to 30 degrees to keep the test time within acceptable limits and to reduce the burden on the patient.

There are occasions when quantification of the extent of the visual field is important, and this can be achieved through a suprathreshold test without excessive test time. For example, clinical trials for novel treatments may want to target specific disease states or require patients’ peripheral visual function to be characterised. For this reason, an adequate test of the peripheral field is required. However, as this may often be a supplementary test, the time spent on this should not detract from the primary outcome measure. Therefore, an evenly spread test grid that does not take too much time would be required. The test must provide a quantitative assessment suitable for longitudinal monitoring or disease state classification. The objective of this paper is to examine if this can be achieved through a modification of the widely used Esterman visual field test.

Since 1984, the Esterman test scoring system has been the American Medical Association standard for rating vision capabilities.^5^ The test examines more than 130 degrees of the binocular field using 120 white test locations shown with equal, non-adjustable suprathreshold Goldmann size III stimuli at an intensity of 10 dB. Missed test points are retested, and a defect is recorded if the second presentation is also missed by the patient. This method allows for naturally occurring binocular enhancement, in which two seeing eyes compensate for defects in the fellow eyes. Thus, a binocular functional measure is obtained which more closely relates to real-world function than monocular testing. The Esterman test visual field test is used in several countries including the UK to assess drivers for significant visual field defects.^6^

**Figure 1:**
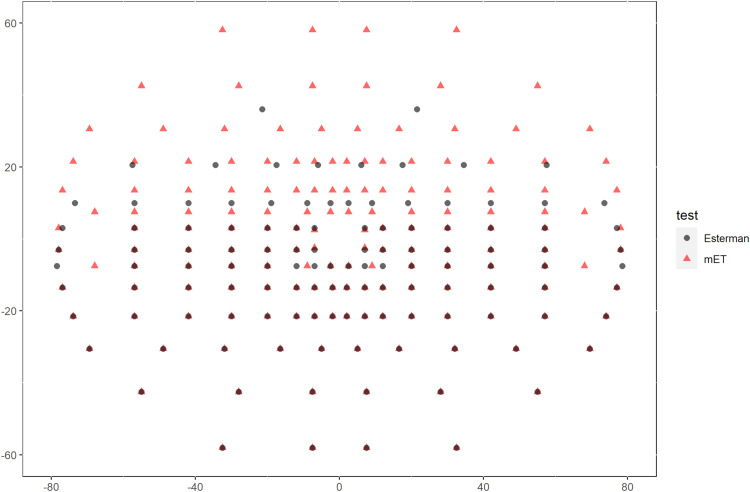
A map of the binocular visual field. Comparison of stimulus locations in the traditional Esterman test (grey circles) and the modified Esterman, mET (red triangles). The brown symbols are overlapping points.

The distribution of test points of the Esterman test was originally developed for scoring of functional impairment.^7^ The test locations are spaced unequally to favour the more functionally valuable parts of the field. This over-represents some areas of the field such as inferior and under-represents other areas such as superior, which can affect the reliability of safety monitoring. The Esterman test has only 2 points above 20 degrees superiorly, which would fail to characterise the 45–63 degrees superior range of normal vision.^8^ We therefore created a modified Esterman test called the modified Esterman (mET) test with an even spread of test points and 2 planes of symmetry across the x and y axis (Figure 1). Additionally, we used the larger Goldman size V stimulus in order to improve the scope for safety monitoring.

We tested the mET test in a group of patients with inherited retinal disease. Two conditions were chosen to represent primary peripheral visual field loss (choroideremia) and primary central visual field loss (Stargardt disease).

Choroideremia is a rod-cone dystrophy characterised by progressive atrophy of the choroid, retinal pigment epithelium (RPE), and photoreceptors. Choroideremia primarily affects male subjects, has an estimated prevalence of 1 in 50,000, and typically presents with nyctalopia and peripheral visual field degeneration.^9^ It is currently being treated with gene therapy in clinical trials (NCT02407678 and NCT03496012).

Stargardt disease is a cone-rod dystrophy causing photoreceptor loss with an estimated prevalence of 1 in 10,000.^10^ A number of therapeutic agents are currently being trialled in patients with Stargardt disease (NCT01367444, NCT 03772665, and NCT03297515). These conditions were chosen due to their relevance to current interventional trials, making this study directly relevant to advances in care of patients with inherited retinal degenerations. Additionally, both diseases have an impact at a similar age so age could be removed as a confounding factor in analysis.

This paper presents the new mET and compares it against the established Esterman test using Voronoi visualisation to establish its utility in inherited retinal degeneration.

## Materials and Methods

### Test Development

Forty additional points extend the field area tested to 60 degrees superiorly allowing a clearer insight into the impact of disease on the visual function (Figure 1). The mET test was designed to present an even spread of test stimuli across the visual field, which is more appropriate for characterising visual field loss for the purposes of disease classification and quantification.

The mET was implemented on the Octopus perimeter (Haag-Streit, Koeniz, Switzerland) using R code with the Open Perimetry Interface (OPI). The OPI was designed to be a simple set of functions that are comprehensible by vision scientists and allows custom tests to be implemented on commercial perimeters.^11–16^ The OPI has been used in a number of settings to develop testing regimes for investigation of the visual system and disease, and to probe the effectiveness of perimetric strategies.

**Figure 2:**
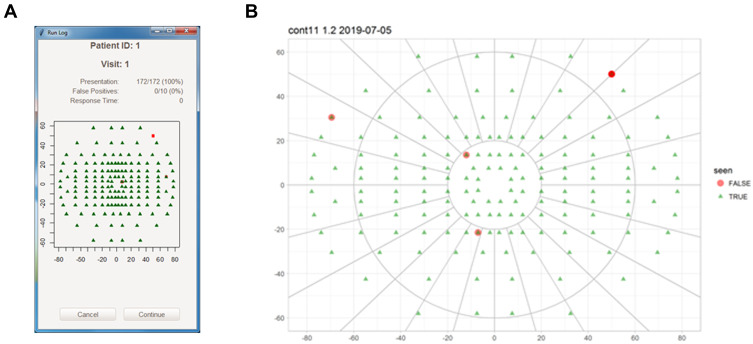
(**A**) Example live output from modified Esterman (mET) showing points seen in green and not seen in red. Number of points presented is also shown as well as false positive responses. (**B**) Example graphical output on completion of the mET.

The mET comprises a Graphical User Interface (GUI) and base script to run the supra-threshold screening algorithm. The mET uses R packages OPI, tcltk2, ggplot2,^17^ labelling, digest, gridExtra, ggforce, and tkrplot which can all be installed via the Comprehensive R Archive Network (CRAN). The GUI is presented to allow the user to intuitively enter patient details and select the desired testing pattern. In line with the guiding principles of the OPI the mET is open-source and free for all to use (https://github.com/andrewscolm/mET). The GUI features real-time graphing of stimulation location and detection (Figure 2A), which allows the mET operator to track the participant responses and make adjustments where necessary. The operator is able to pause or cancel the test during the examination to give the participant further instruction or a comfort break.

The output files are a tiff image of the test pattern and responses (Figure 2B); a csv log file containing the x and y coordinates of each stimulation, the stimulation size and level and whether or not the stimulation was seen; and a summary csv detailing the number of failed false positives, the number of stimulations with a positive response the first try, the number of stimulations with a positive response on the retest and the number of test locations with a recorded defect (not seen).

In order to maximise the dynamic range of the test, the larger Goldmann V (64 mm^2^ or 1.72 degrees) stimulus size was selected instead of the usual, Goldmann III stimulus (4 mm^2^ or 0.43 degrees). By adding forty additional points, the field was extended to 60 degrees superiorly (Figure 1). The testing paradigm copies that employed by the Esterman with each location test repeated if not seen the first time, in a suprathreshold manner with a size V white stimulus at an intensity of 10 dB. A defect is recorded if the retest results in a negative response and if the target is seen once and not seen once, this is also shown. Table 1 summarises the test similarities and differences.

**Table 1:** Test Characteristics and Parameters Highlighting the Similarities and Differences Between the Original Esterman Test and the Modified Esterman (mET) Test. The Parameters That are Identical are in Cells That Have a Light Grey Background

Test Characteristic	Original Esterman	Modified Esterman (mET)
Mode of testing	Binocular	Binocular
Thresholding	Suprathreshold, fixed intensity of 10dB	Suprathreshold, fixed intensity of 10dB
Visual field area tested	Full field	Full field
Number of points tested	120	160
Stimulus presentation	Each point tested twice	Each point tested twice
Size of stimulus	Goldman size III	Goldman Size V
Spread of points tests	Focussed inferiorly	Spread evenly across the visual field, including the superior field
Speed of testing	Quick	Quick
Purpose of testing	Evaluating functional vision	Disease classification and quantification

### Patient Testing

Participants with choroideremia and Stargardt disease, and age- and sex-matched controls were tested as part of an ongoing study investigating the impact of visual field loss on the visual brain. Participants were aged 17–70 years, with no additional ophthalmic conditions, and a minimum vision of hand movements. This study is approved by the Health Regulatory Authority (17/LO/1540) and adheres to the Declaration of Helsinki. All participants provided informed consent. All participants underwent both the standard Esterman test and mET tests twice. Each test took 3–6 minutes to complete and breaks were provided as needed. The severity of field loss (normal/mild/moderate/severe) was graded using the average of the two Esterman and the average of the two mET tests, to allow for a possible learning effect following the first test.

### Analysis of Test Results

#### Voronoi Tessellation

Results were analysed using Voronoi tessellation.^18^ In brief, Voronoi tessellations partition the surface into regions so that the centre of each cell is its mean (centre of mass). Every point in a given Voronoi polygon is closer to its generating point than to any other cell so interpolation is not required.

#### Disease State Classification

A “normal” test result was defined as when no test locations were abnormal, a result with “mild disease” was defined by a few scattered abnormal locations, “moderate disease” was defined as at least 2 clusters (with at least 3 damaged locations) to <50% locations not seen, and “severe” was defined when ≥50% locations were missed. Percentage of test points seen was calculated using open source code (https://github.com/jkjolly/Octopus-periphery-score) developed in MATLAB (version 2018a, MathWorks Inc., Natick, MA, USA).

### Statistical Analysis

The agreement between severity of field loss graded from the Esterman and mET tests was assessed using weighted kappa statistics. Retest analysis consisted of Bland-Altman analysis between Esterman and mET. Repeatability was analysed using the repeatability coefficient for the Esterman test and for mET.^19^ All analyses were conducted in R^20^ and figures were produced using the package ggplot2^17^.

### Participants

Subject demographics are shown in Table 2. Sixty-two participants underwent assessment (mean age 38 years). This comprised 19 choroideremia patients (mean age 39 years), 17 Stargardt patients (mean age 37 years) and 26 control participants (mean age 39 years). Compliance with the mET was good, and no participant had a false-positive rate above 20%.

**Table 2:** Subject Demographics

	N	Female (%)	Age (Mean (SD))	No. Smokers (n=)	Disease Severity According to Visual Field Loss	Concomitant Health Problems (n=)
Choroideremia	19	11 (male phenotype)	38.9 (13.9)	0	Mild=6 Mod=8 Sev=5	Autoimmune condition=2 IBS=2 Endometriosis=1 Gout=1 OCD=1 Heart problem=1 Asthma=1
Stargardt	17	59	37.4 (16.8)	1	Mild=3 Mod=8 Sev=6	Sinus problem=1 Diabetes=1 Autoimmune condition=1 Asthma=1 Migraine=1 Chronic pain=1 Coeliac=1
Control	26	42	38.5 (14.5)	0	N/A	Heart problem=1 ADHD=1 Prostate problem=1 Chronic pain=2 Autoimmune disease=1 Asthma=1
Overall	62	37	38.4 (14.7)	1		

## Results

**Figure 3:**
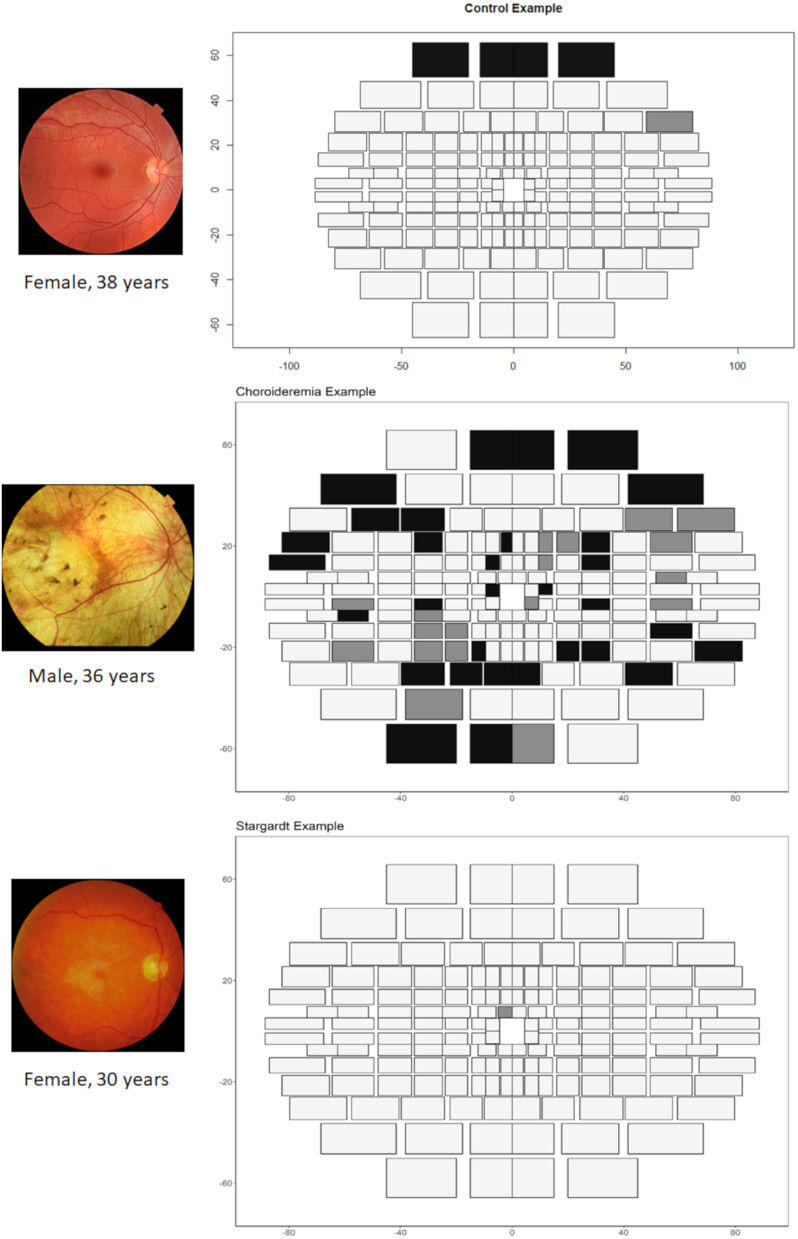
Representative examples of the output for the three participant groups from the modified Esterman test with corresponding fundus pictures to demonstrate the disease phenotype. Details of the participant sex and age provided. Black represents locations not seen, dark grey locations were seen on one presentation and light grey locations were seen on both locations.

Representative examples of each participant population are shown in Figure 3. The size of the boxes is representative of the distance to neighbouring points and hence becomes larger towards the periphery, particularly in the vertical meridian. Black indicates that the point was not seen on either presentation. Dark grey points were seen on a single presentation and light grey points were seen on both presentations. The lid artefact present in the control participant was commonly seen, visible in 18/9 field plots in the absence of any other field defects. This indicates that the top three to six points were neither informative nor strictly necessary and can be removed in future tests.

**Figure 4:**
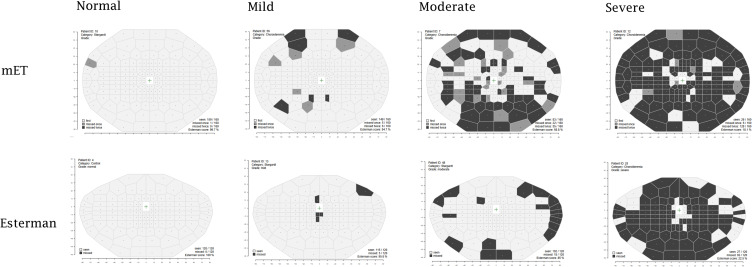
Voronoi tessellation visualisation comparing the Esterman and mET test paradigms across disease severity classifications. Black represents locations not seen, dark grey locations were seen on one presentation and light grey locations were seen on both locations.

**Figure 5:**
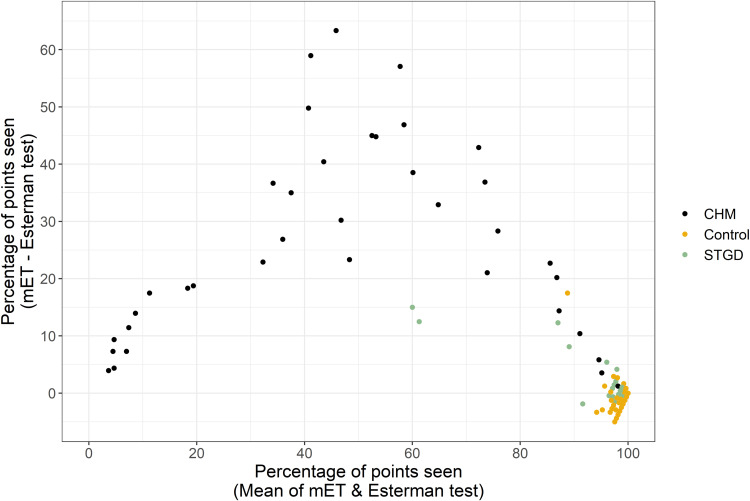
Plot showing the limit of agreement distribution comparing Esterman and mET tests for choroideremia, Stargardt and control patients.

**Figure 6:**
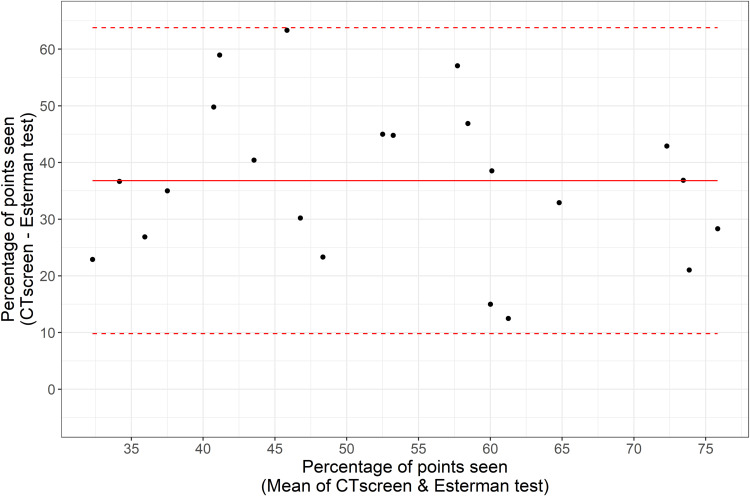
Bland Altman plot showing the limit of agreement comparing Esterman and mET tests for the percentage of points seen between 20% and 80%.

Figure 4 shows examples of Voronoi tessellation demonstrating the disease severity states as reported by both the Esterman and mET tests. The Voronoi tessellation shows that mET is able to show up far more information about areas of visual field loss, and is therefore more useful for safety monitoring. Due to the severe non-parametric distribution of points (Figure 5) limit of agreement analysis was restricted to percentage of points seen across the mET and Esterman test of between 20% and 80%. For fields with average points seen across the mET and Esterman test of between 20% and 80% there is a mean systematic difference of 27% (Figure 6). This means that the mET determines over a quarter more test locations to be defective compared to the Esterman test, many of them in superior locations. This difference is present in the choroideremia cohort but not the Stargardt or control cohorts, who have well preserved peripheral visual fields. If there was no additional information gained by using the mET, then the agreement between grading using the mET and grading using Esterman test would be expected to be close to unity. The agreement between the Esterman and mET was moderate in the control cohort (κ=0.59, 95% CI: 0.38−0.79) and the choroideremia cohort (κ=0.41, 95% CI: −0.09–0.92). There was poor agreement for the Stargardt cohort (κ=0.14, 95% CI: −0.50–0.77). The overall agreement was moderate (κ=0.48, 95% CI: 0.29–0.67) (Table 3).

**Table 3:** Cohen’s Kappa Correlation Estimates

Cohort	Kappa Estimate	Lower 95% Confidence Interval	Upper 95% Confidence Interval	Interpretation
Choroideremia	0.41	−0.09	0.92	Moderate
Stargardt	0.14	−0.50	0.77	Poor
Control	0.59	0.38	0.79	Moderate
Combined	0.48	0.29	0.67	Moderate

## Discussion

We present a new open source test to be used for the assessment of the peripheral visual field. It is a suprathreshold test that is able to quantify the binocular extent of vision loss quickly using a Goldmann size V stimulus once or twice depending on the subject’s responses. The coverage of the visual field with a symmetrical arrangement of test locations allows the characterisation of retinal disease presenting with different patterns of visual field loss. The mET comprises a base R script to run supra-threshold screening algorithms, a Graphical User Interface and a testing pattern. It utilises the OPI initiative and can therefore be implemented locally by others on their devices without the requirement for additional hardware. This makes this test suitable for implementation as part of multinational multicentre clinical trials using any equipment that supports open-source implementation.

We found low overall agreement between field loss grading using the Esterman and mET tests, thereby confirming that the additional points on mET provide useful information. The difference between the Esterman and mET was most marked in patients with choroideremia, as would be expected, since it manifests with peripheral visual field loss.^9^ The extra test locations yield a significant increase in information about disease severity, allowing for a finer grading compared to what can be achieved with the Esterman. This provides more appropriate information for longitudinal quantitative grading of disease progression which can be used as a safety measure in clinical trials. The original Esterman test was weighted for the lower visual field so few points represent the inferior retina. Although this may be useful for functional testing, this makes it less useful for safety testing due to the asymmetrical representation of the retina. The mET therefore corrects this deficiency by providing a balanced representation of the visual field. In patients with Stargardt disease, visual field loss is localised in the central area,^21^ which is not well represented by suprathreshold tests. Increasing the extent of screening of the peripheral visual field will therefore not provide additional information about disease progression until visual loss is extreme. Interestingly, the kappa agreement between tests was lowest in the Stargardt patient group. This may be a reflection of the increased variability inherent at the border of the scotoma in the central area, which may move patients between mild and moderate disease categories without providing more information about the nature of the visual field loss. However, kappa agreement is dependent on the base rate of score incidence and may be less useful with the narrower range of presentation of visual deficits.

The 160-point test pattern delivers a standardised method of characterising visual field loss, with equal weighting across the peripheral visual field. The top 3–6 points can be removed without affecting test utility as they are heavily influenced by eyelid position. Visual field tests involve a trade-off between test time and specificity. Adding an additional 40 points results in a better topographical mapping of the visual field loss without a large increase in test time. This makes the test useful for a number of scenarios and for quantitatively monitoring a wider area of retinal function. Importantly, the reliability of the test is not compromised with the additional points.

The vast majority of visual field research has been conducted in the area of glaucoma with relatively little attention paid to inherited retinal degeneration. Recent attention to suprathreshold testing in glaucoma has shown it has value in a diagnostic capacity with gains in test times.^22^

With the rise of clinical trials taking place to treat previously untreatable retinal conditions,^23^ it is important to adequately screen patients for inclusion into these trials. Rod-cone dystrophies present with peripheral visual field loss from the earliest stages and therefore can be quickly classified using peripheral visual testing. The mET forms a useful adjunct test to threshold of the central visual field in order to quantify a large area of the visual field. The mET presents a viable solution, which is quick and easy to administer on the commercially available Octopus perimeter. Additionally, the periphery must be monitored even when treatment zones are in the central retina to provide a safety measure of toxicity reactions in the peripheral retina. This method provides a quick, repeatable quantitative safety measure than can be implemented across multiple test centres and will likely require less training required than traditional kinetic or thresholding tests.

## Conclusion

Compared to the widely used Esterman test, the mET provides additional test locations, particularly in the superior field, which are critical for the accurate characterization of visual defects in the presence of visual field loss. This additional information cannot be extrapolated from the original Esterman test or from testing the central visual field alone.

## Abbreviations

GUI, graphical user interface; mET, modified Esterman; OPI, open perimetry interface; RPE, retinal pigment epithelium.
